# Functional Foods and Health Promotion

**DOI:** 10.3390/nu18061000

**Published:** 2026-03-21

**Authors:** Hammad Ullah, Marco Dacrema

**Affiliations:** 1 School of Pharmacy, University of Management and Technology, Lahore 54000, Pakistan; 2 Department of Pharmacy, University of Napoli Federico II, 80131 Naples, Italy; marcodacrema1991@gmail.com

Chronic diseases, also known as non-communicable diseases (NCDs), are long-lasting conditions characterized by slow progression. They arise from a combination of genetic, physiological, behavioral, and environmental factors. According to the World Health Organization (WHO), NCDs are responsible for approximately 71% of all deaths worldwide each year, making them the leading cause of mortality. The four major NCDs are cardiovascular diseases (17.9 million deaths annually), cancer (9.0 million), respiratory diseases (3.9 million), and diabetes (1.6 million) [1]. The primary risk factors for NCDs include unhealthy diets, physical inactivity, tobacco use, and harmful alcohol consumption. As these conditions often develop gradually from early life due to lifestyle-related factors, a substantial proportion of NCDs are preventable through timely interventions and healthy behavioral modifications [2].

Functional foods are defined as foods that provide health benefits beyond basic nutrition. Although numerous definitions exist worldwide, there is no single official or universally accepted definition. From one perspective, all foods may be considered functional because they contain nutrients and exert physiological effects on the body. However, in practice, the term “functional food” is often used in marketing to describe products whose appeal is based on their perceived or promoted health benefits. Some critics even argue that any product can be labeled as functional if it is marketed effectively, particularly when accompanied by specific health claims. A widely cited working definition was proposed by the European Commission through its Concerted Action on Functional Food Science in Europe (FUFOSE). According to FUFOSE, a functional food is *“a food that beneficially affects one or more target functions in the body beyond adequate nutritional effects, in a way that is relevant to an improved state of health and well-being and/or reduction of risk of disease. It is consumed as part of a normal food pattern. It is not a pill, a capsule, or any form of dietary supplement.”* [3]. Other organizations have proposed similar definitions. Health Canada defines functional foods as those that resemble conventional foods, are consumed as part of the usual diet, and have demonstrated physiological benefits or reduce the risk of chronic disease beyond basic nutritional functions. The International Food Information Council (IFIC) describes them as foods or dietary components that may provide health benefits beyond basic nutrition. Likewise, the International Life Sciences Institute of North America (ILSI North America) defines functional foods as those that, by virtue of physiologically active components, offer health benefits beyond basic nutritional value [4].

Food bioactive compounds are naturally occurring nutrients and non-nutritive substances that exert biological effects in the body, ideally contributing to the promotion of health. These compounds are typically present in small quantities in functional foods, and research on their effects in humans is still evolving [5]. Epidemiological evidence suggests that a high intake of naturally occurring functional foods, particularly fruits and vegetables rich in bioactive constituents is associated with a reduced risk of chronic diseases such as obesity, type 2 diabetes mellitus, metabolic syndrome, cardiovascular diseases, and cancer [6]. Various natural bioactive compounds, including herbal extracts, micronutrients, polyphenols, phytosterols, dietary fibers, prebiotics, and probiotic strains, have been investigated for their potential direct and indirect interactions with the molecular pathophysiological mechanisms underlying chronic diseases. Nevertheless, further well-designed preclinical and clinical studies are required to firmly establish their efficacy, safety, and tolerability.

This research on “Functional Foods and Health Promotion” aims to identify, develop, and establish functional foods by investigating the biological mechanisms through which food bioactive components exert their effects, ultimately improving health outcomes. The study specifically addresses gaps in functional food research by integrating mechanistic insights, translational validation, and clinical evidence, thereby strengthening the scientific basis for nutrition-based prevention of NCDs. A total of five studies were included in this research: one clinical trial, two original research articles, and two literature reviews.

Song et al. [7] investigated the immunomodulatory effects of *Lactococcus lactis* OTG1204 and its underlying molecular mechanisms in RAW 264.7 macrophages. Their study reported that OTG1204 restored body weight, spleen, and mesenteric lymph node indices, enhanced natural killer cell activity, and normalized populations of innate and adaptive immune cells. It also promoted T cell activation and cytokine secretion in cyclophosphamide-treated mice. Furthermore, OTG1204 improved gut barrier integrity by upregulating mucin 2 and tight junction proteins, and modulated the gut microbiota by restoring the Firmicutes/Bacteroidetes balance while reducing the abundance of *Actinobacteria* and *Tenericutes*. Baek et al. [8] demonstrated that eriocitrin, a flavanone present in peppermint and citrus, inhibits angiogenesis by suppressing endothelial cell growth and migration and inducing apoptosis. At the molecular level, eriocitrin regulated the MAPK/ERK pathway and VEGFR2, inhibited the downstream PI3K/AKT/mTOR signaling pathway, and reduced the expression of MMP-2 and MMP-9, effectively blocking the processes required for new blood vessel formation. Buccato et al. [9] evaluated the hypoglycemic effects of a food supplement containing *Zea mays* L., *Gymnema sylvestre* (Retz.) R.Br. ex Sm, zinc, and chromium in a monocentric, randomized, double-blind, placebo-controlled clinical trial. After three months of supplementation, the intervention significantly reduced fasting blood glucose and glycated hemoglobin (HbA1c), suggesting that this food supplement may improve glucose homeostasis in individuals with mildly impaired fasting glucose by enhancing glucose metabolism.

A review by Stellaard and Lütjohann [10] concluded that phytosterol supplementation (2–3 g/day) reduces LDL-cholesterol (LDL-C) absorption, leading to an average LDL-C reduction of approximately 10%. However, emerging evidence indicates that increased phytosterol intake may, in certain cases, contribute to atherosclerosis. This effect appears to depend largely on individual genetic polymorphisms in NPC1L1 and ABCG5/G8 transport proteins, as well as the overall risk reduction achieved through LDL-C lowering. Lee and Choi [11] reviewed the biological and pharmacological properties of *Lycium ruthenicum* Murray (commonly known as black goji berry or black wolfberry), highlighting its antioxidant, anti-inflammatory, anti-radiation, immune-enhancing, anti-tumor, and protective effects.

In conclusion, this research topic explored the potential health benefits of functional food ingredients, with a particular focus on their molecular mechanisms of action. These ingredients have been associated with improvements in systemic inflammation, glucose and lipid metabolism, carcinogenesis, and modulation of the gut microbiota. Although further research is required to fully elucidate their long-term effects and underlying mechanisms, functional food ingredients represent a promising component of a balanced diet, offering a natural strategy to support overall health and well-being. The following directions are suggested for future research:

1. Multi-omics approaches to uncover the complex interactions among bioactive compounds, the gut microbiome, and host signaling pathways.
2. Systems-level analyses to enable deeper mechanistic validation of health claims and enhance reproducibility across populations.
3. Large-scale, multi-center clinical trials to confirm efficacy, assess long-term safety, and determine optimal dosing strategies.
4. Regulatory harmonization across international frameworks to translate scientific evidence into effective public health recommendations.
